# Cellular miRNAs and viruses: trends in miRNA sequestering and target de-repression

**DOI:** 10.1128/jvi.00914-25

**Published:** 2025-06-18

**Authors:** Bonita H. Powell, Kenneth W. Witwer, Mollie K. Meffert

**Affiliations:** 1 Solomon H. Snyder Department of Neuroscience, Johns Hopkins University School of Medicine 1466, Baltimore, Maryland, USA; 2 Department of Molecular and Comparative Pathobiology, Johns Hopkins University School of Medicine 1500, Baltimore, Maryland, USA; 3 Department of Neurology, Johns Hopkins University School of Medicine 1500, Baltimore, Maryland, USA; 4 Department of Molecular Biology and Genetics, Johns Hopkins University School of Medicine 1500, Baltimore, Maryland, USA; Universiteit Gent, Merelbeke, Belgium

**Keywords:** microRNA, miRNA, Argonaute, AGO, viruses, HCV, AGO-CLIP, CLEAR-CLIP, RNA-seq

## Abstract

Altered gene regulation downstream of infection has been linked to devastating cancers and neurological diseases, highlighting the importance of understanding viral:host gene interactions. Historically, approaches based on bioinformatic binding prediction showed that host microRNAs (miRNAs) can target and regulate viral genes to impact viral replication and pathogenesis. More recently, Argonaute cross-linking and immunoprecipitation (AGO-CLIP) and advancements incorporating a miRNA:target RNA ligation step (AGO-CLIP + ligation) enable a global view of miRNA interactions with target cellular and viral transcripts. These genome-wide approaches paired with RNA sequencing reveal that miRNA binding to viral transcripts can not only act conventionally to regulate viral replication but can also act to reduce miRNA targeting of host genes with resulting de-repression of host target genes and downstream biological impacts. Viruses with accumulated evidence of miRNA sequestration are selected as examples for review and include hepatitis C virus (HCV), severe acute respiratory syndrome coronavirus 2 (SARS-CoV-2), and respiratory syncytial virus (RSV). The significant impact of target de-repression on host cellular biology warrants a broader investigation of this mechanism. In this mini-review, we examine examples of crosstalk between host miRNAs and viral transcripts and highlight the advance and potential of analyses from AGO-CLIP + ligation with RNA-seq for expanding the identification of global miRNA:viral target interactions and interrogating the biological impacts of host miRNA sequestering and target de-repression. Host target de-repression by miRNA:viral target interactions could shed light on antiviral therapeutic candidates to aid in mitigating consequences such as malignancies and neurodegeneration.

## INTRODUCTION

MicroRNAs (miRNAs) are short (averaging 22 nucleotides) non-coding RNAs that regulate the expression of protein-coding genes at the post-transcriptional level. Since the discovery of miRNAs in 1993 (1, 2), critical regulatory roles of miRNAs in tissue development and cellular homeostasis have been defined (3–6). As with the expression of protein-coding genes, miRNA gene expression varies according to tissue type (7–10). In some instances, miRNAs regulate protein translation to govern cellular differentiation, proliferation, and apoptosis in a tissue type- or cell type-specific manner. MiRNAs also have critical roles in human disease pathology, with the expression levels and functions of miRNAs often dysregulated in diseases including cancer, neurodegeneration, heart disease, and viral infection (6, 11–16).

MiRNA genes are typically transcribed by polymerase II as primary miRNA (pri-miRNA) transcripts that are cleaved by the type III RNase, Drosha, and processed into a hairpin-like precursor miRNA (pre-miRNA) molecule (17–19). Pre-miRNAs are then transported from the nucleus to the cytoplasm for subsequent cleavage by another type III endonuclease, DICER, into a double-stranded duplex (20). One strand of the duplex, either the 5 p or 3 p strand, is then loaded onto an RNA binding protein, Argonaute (AGO) (21). Humans express four AGO proteins; AGO2 is the most abundant form (22, 23). AGO that is associated with mature single-stranded miRNAs is a core component of the RNA-induced silencing complex (RISC) (5). Generally, RISCs regulate gene expression by binding to the 3’ untranslated regions (3’UTRs) of messenger RNAs (mRNAs) or to other mRNA regions and reduce the expression of targeted transcripts by inhibiting mRNA translation and inducing mRNA degradation (5, 24). The RISC-binding site within a target mRNA has partial complementarity with the guide miRNA; at a minimum, pairing within the miRNA seed region, miRNA nucleotide positions 2-7, with a target mRNA is required (5). Furthermore, miRNA seed binding can include base pair mismatches, bulges, and G∙U wobble base pairing that is often flanked by compensatory pairing near the 3’ end of the miRNA (5, 24).

Humans encode between 500 and 1800 miRNA genes (5, 25), according to the MirGeneDB manually curated database of validated metazoan species miRNAs (26, 27) or miRbase (28). Given the short miRNA length and minimal required base-pairing with an mRNA, an individual miRNA can have many possible targets (24). MiRNAs that belong to families that share the same seed sequence or to gene clusters that are transcribed together often have similar biological functions (5, 24). In addition, both DNA and RNA viruses can encode miRNAs. The first virally encoded small RNA, simian 40 (SV40)-associated small (SAS) RNA with functions similar to miRNA was identified in 1980 and shown to bind with perfect complementarity and regulate early SV40 transcripts (29, 30). SV40 (30), several alpha, beta, and gamma herpesviruses (31), and severe acute respiratory syndrome coronavirus 2 (SARS-CoV-2) (32) are also known to encode miRNAs that regulate viral and host transcripts. Functions of virally encoded miRNAs and miRNA-like small RNAs in viral latency regulation and host immune suppression have been recently reviewed elsewhere and will not be the focus of this review (33–35).

The complexity of miRNA:target RNA interactions has made investigating all possible miRNA and miRNA family interactions difficult to experimentally validate. Useful bioinformatic tools such as miRanda (36), DIANA (37), TargetScan (38), and RNAhybrid (39) have helped predict miRNA target binding (40). For example, TargetScan is a widely used tool that identifies 6-mer (miRNA seed positions 2–7), 7-mer (miRNA seed positions 2–7 + position 8), and target 8-mer (miRNA seed positions 2–8 followed by an ‘A’) binding sites within mRNA 3’UTRs and has greatly improved in predicting targeting efficacy over the past ~20 years (38). However, predictive algorithms can produce numerous false-positive and false-negative results, as well as being typically limited to 3’ UTRs and overlooking unconventional miRNA binding to 5’ untranslated regions (5’ UTRs), introns, and coding sequences (CDS). Additionally, these *in silico* predictive tools do not include viral transcripts, which would allow the prediction of human miRNA:viral transcript binding interactions to facilitate the understanding of viral and host miRNA functional interactions.

Early methods for predicting miRNA binding within viral genes typically included the selection of candidate miRNAs, followed by scanning the viral genome for potential miRNA binding sites and calculating the minimum free energy of hybridization for potential miRNA:viral target RNA sequence alignments. As detailed in the subsequent section, this approach identified multiple predicted binding sites for miR-122 within the hepatitis C virus (HCV) genome. MiR-122 is required to promote HCV translation and replication in the liver through its 5’UTR binding site (16). In contrast, miR-29, miR-28, miR-125b, miR-150, miR-223, and miR-382 were found to inhibit human immunodeficiency virus (HIV) through interactions with viral mRNA 3’UTRs in immune cells (41, 42). Additionally, miR-323, miR-491, and miR-645 inhibit the 2008 H1N1 influenza A virus by interacting with a shared (GUGG) binding sequence that is conserved across several influenza strains (43), and miR-1293 inhibits Kaposi’s sarcoma-associated herpesvirus (KSHV) by binding and suppressing the viral interleukin-6 transcript (44). Similarly, identification of potential miRNA binding sites was used to show that miR-142, miR-296, miR-28, and miR-22 could bind and inhibit Eastern equine encephalitis virus (EEEV) (45, 46), enterovirus 71 (EV71) (47, 48), human T cell leukemia virus type 1 (HTLV-1) (49, 50), and coxsackie virus B3 (51, 52), respectively (Table 1). Together, these candidate-based approaches have demonstrated multiple host miRNA:viral target RNA interactions in which the miRNA typically functions in a conventional target-suppressing manner.

**TABLE 1:** Human pathogenic viruses regulated by cellular miRNAs^*a*^

Human virus	Genome	miRNA	Method	Citation no., year
Non-AGO CLIP approach				
Hepatitis C virus (HCV)	RNA	miR-122	Binding prediction/mutational analysis	16, 2005
Human immunodeficiency virus (HIV)	RNA	miR-28, miR-125b, miR-150, miR-223, miR-382	Binding prediction/mutational analysis	41, 2007
Human immunodeficiency virus (HIV)	RNA	miR-29a	Binding prediction/ anti-miRNAs	42, 2008
Influenza A virus (IAV)	RNA	miR-323, miR-491, miR-654	Binding prediction/ mutational analysis	43, 2010
Kaposi’s sarcoma-associated herpesvirus (KSHV)	DNA	miR-1293	Binding prediction/ mutational analysis	44, 2011
Eastern equine encephalitis virus (EEEV)	RNA	miR-142	Binding prediction/ mutational analysis	45, 2013
Enterovirus 71 (EV71)	RNA	miR-296	Binding prediction/ mutational analysis	47, 2013
Human T cell leukemia virus type 1 (HTLV-1)	RNA	miR-28	Binding prediction/ mutational analysis	49, 2015
Coxsackie virus B3	RNA	miR-22	Binding prediction/mutational analysis	52, 2024
AGO-CLIP approach				
Herpes Epstein Barr virus (EBV)	DNA	miR-17/92	AGO-CLIP	53, 54, 2012
Herpes simplex virus 1 (HSV-1)	DNA	miR-138	AGO-CLIP	55, 2014
Hepatitis C virus (HCV)	RNA	miR-122	AGO-CLIP	56, 2015
Herpes simplex virus 2 (HSV-2)	DNA	miR-138	AGO-CLIP	57, 2022
AGO-CLIP + ligation				
Hepatitis C virus (HCV)	RNA	miR-122	AGO-CLIP + ligation	51, 2016
Hepatitis B virus (HBV)	DNA	miR-15/16	AGO-CLIP + ligation	51, 2016
Poliovirus	RNA	miR-122, miR-92, miR-320	AGO-CLIP + ligation	51, 2016
Coxsackie virus (CVB)	RNA	miR-92	AGO-CLIP + ligation	51 2016
Sindbis virus (SINV)	RNA	miR-27, miR-122, miR-15, miR-92	AGO-CLIP + ligation	51, 2016
Chikungunya virus (CHIKV)	RNA	miR-21, miR-181, miR-122	AGO-CLIP + ligation	51, 2016
Venezuelan equine encephalitis virus (VEEV)	RNA	miR-15, miR-92	AGO-CLIP + ligation	51, 2016
Severe acute respiratorysyndrome coronavirus 2 (SARS-CoV-2)	RNA	Let-7a, miR-29a, miR-17, miR-27a, miR-30a, miR-15	AGO-CLIP + ligation	58, 2023
Respiratory syncytial virus (RSV)	RNA	miR-26, miR-27	AGO-CLIP + ligation	59, 2024

^*a*^ Included are human pathogenic viruses with human miRNA interactions reported to bind and regulate viral genes by: *in silico* prediction analysis, AGO-CLIP followed by *in silico* predictions, and by AGO-CLIP + ligation. Note that non-human pathogenic viruses and virally encoded miRNAs are not included in this table.

In addition to target gene regulation, host miRNA:viral target interactions can induce host miRNA degradation to inhibit miRNA function and enhance viral replication. Virally induced miRNA degradation is mediated by miRNA decay elements (miRDEs), which are transcripts derived from some viral genomes that function in a sequence-specific manner to trigger host miRNA degradation (60). MiRDE is broadly defined, and the extent to which it shares molecular mechanisms with target-derived miRNA degradation (TDMD) (61) remains unclear. Examples of miRDE include miR-27 degradation by the herpesvirus saimiri (HVS) small nuclear RNA miRDE, HSUR1, in T-cells (62, 63), miR-27 degradation by the murine cytomegalovirus (mCMV) miRDE transcript m169 (64), and degradation of miR-17-92 cluster miRNAs, miR-17 and miR-20a, by a human cytomegalovirus (HCMV) intergenic sequence-derived miRDE to increase HCMV replication (60). MiRNA:viral target interactions have also been shown to inhibit host miRNA biogenesis and alter viral replication. For example, the EBV-encoded miRNA, miR-BART6-5p, blocks miRNA processing and maturation; miR-BART6-5p has multiple binding sites within the 3’UTR of Dicer mRNA and functions to inhibit Dicer translation and global processing of Dicer-dependent precursor miRNAs to accelerate viral replication (65). Biologically significant interactions with host miRNA biogenesis in which the miRNA is virally encoded have also been documented. These include the human herpesvirus 6A (HHV-6A) encoded miRNA, miR-aU14, which selectively inhibits the primary miRNA transcript processing of host miR-30 family members by direct loop binding to promote reactivation from latency (66). KSHV-derived miRNAs, miR-4-3, miR-6-3p, miR-7, and miR-10a, can bind directly to the 3’UTR of MCP-1-induced protein 1 (MCPIP1) and lower its expression. MCPIP has an RNase function and can cleave the terminal loops of precursor miRNAs, which prevents processing by Dicer and promotes degradation of host and viral miRNAs (67).

Although binding prediction and candidate-based approaches have yielded valuable insights into the multiple ways that miRNA:target RNA interactions can impact viral replication and host cell biology, selecting individual miRNAs of interest for analysis and functional follow-up from the large number of human miRNA species has been limiting. In the early 2010s, high-throughput methods to identify transcriptome-wide RISC binding sites, such as high-throughput sequencing of AGO-associated RNA isolated by crosslinking immunoprecipitation (HITS-AGO-CLIP) (68) and photoactivatable ribonucleoside-enhanced crosslinking and immunoprecipitation (AGO-PAR-CLIP) (69), herein referred to AGO-CLIP, were developed. These techniques both entail ultraviolet (UV) light irradiation of cells or tissues to covalently cross-link nucleic acids to binding proteins *in vivo* with the goal of cross-linking RISCs to target mRNAs for subsequent AGO immunoprecipitation (IP), sequencing of associated RNAs, and miRNA:target hybridization prediction (70). The AGO-CLIP method has been applied to identify miRNA-mediated AGO binding to both viral and host RNAs. An exemplary interaction includes the repression of Epstein Barr virus (EBV) genes latent membrane protein 1 (LMP1) and BamHI-fragment H rightward open reading frame 1 (BHRF1) by both the host miR-17 family as well as by EBV-derived miRNAs (53, 54). Herpes simplex virus 1 (HSV-1) and herpes simplex virus 2 (HSV-2) infected cell protein 0 (ICP0) (55, 57, 71) viral transcripts and host genes octamer-binding transcription factor 1 (Oct-1) and forkhead box c1 (FOXC1) (71) binding interactions for viral genome activation (Table 1).

**Fig 1:**
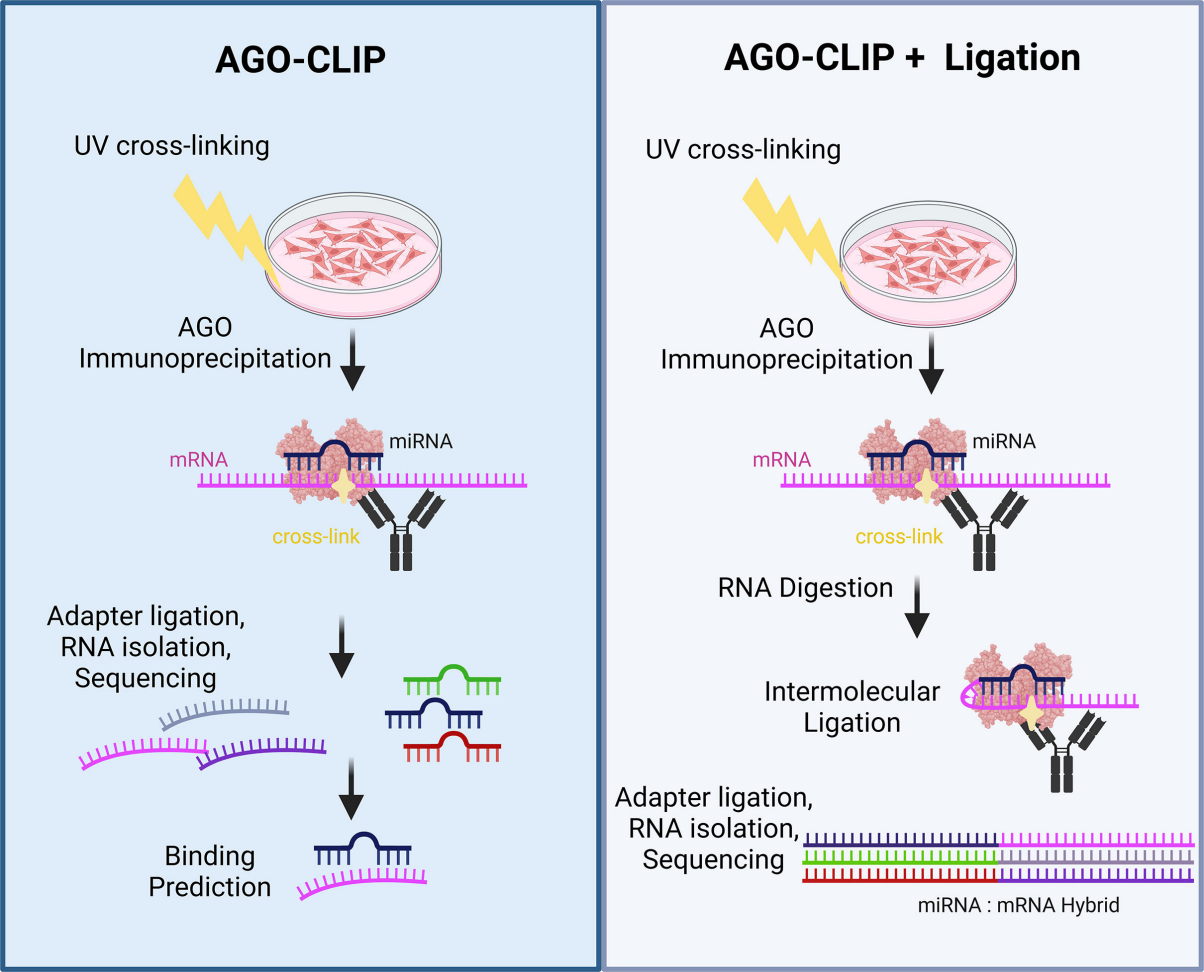
Overview diagram of AGO binding analysis approaches. AGO-CLIP and AGO-CLIP + ligation methods both include ultra-violet light irradiation to covalently cross-link RISCs to target mRNA transcripts. Subsequent steps include RISC immunoprecipitation using anti-AGO antibodies, RNase digestion, etc. The AGO-CLIP + ligation method incorporates a miRNA:mRNA intermolecular ligation step prior to adapter ligation, RNA isolation, and high-throughput sequencing. Bioinformatic analysis of AGO-CLIP sequences employs binding prediction tools to determine miRNA:target interactions; AGO-CLIP + intermolecular reads are analyzed by aligning chimeric reads to miRNA and mRNA sequences.

Although AGO-CLIP provides a genome-wide view of miRNA-mediated AGO binding to both viral and host transcripts, AGO-CLIP methods initially did not identify direct miRNA target interactions and still required prediction tools to locate the potential miRNA seed binding regions within AGO cross-linked transcript data sets. AGO-CLIP approaches have now evolved into several techniques that include a step consisting of an intermolecular ligation between the AGO-bound miRNA and target RNA, generating a chimeric or hybrid miRNA:target RNA molecule (Fig. 1) (Table 1) (72–76). Importantly, these direct ligation approaches have facilitated the discovery of unpredicted interactions and a semiquantitative understanding of miRNA:target RNA interactions. AGO-CLIP methods that include miRNA:target ligation indicate that miRNAs also bind to protein coding sequence (CDS) regions or 5’UTRs of target mRNA, which may also participate in target repression. Several studies used these direct ligation methods to reveal previously unappreciated binding of human miRNAs to viral transcripts. In 2013, cross-linking ligation and sequencing of hybrids (CLASH) was developed as the first approach to directly identify global miRNA:target RNA binding in human cells (Fig. 1) (Table 2) (72). CLASH is an AGO-CLIP + ligation method that involves (i) doxycycline-induced overexpression of protein A-6xhistidine tagged AGO1, (ii) UV irradiation of cells to cross-link RISCs to target RNAs, (iii) tandem immunoprecipitation of tagged AGO1 and bound RNAs, and (iv) a series of enzymatic reactions to digest, repair, and subsequently ligate AGO-bound miRNAs to target RNAs (72). CLASH is the first high-throughput approach to provide direct genome-wide miRNA: target RNA interactions. CLASH also confirmed non-canonical miRNA interactions beyond the 3’UTR of mRNA, which can occur within the CDS and intronic regions (72). After CLASH, similar AGO-CLIP + ligation methods were developed that immunoprecipitate (IP) endogenous AGO proteins rather than an inducible tagged version (Table 2) (73–76). AGO-CLIP + ligation methods have now begun to be used to validate and investigate host and viral miRNA target interactions.

**TABLE 2:** AGO-CLIP +ligation methods^*a*^

AGO-CLIP + ligation methods	Citation no., year
Cross-linking ligation and sequencing of hybrids (CLASH)	72, 2013
Covalent ligation of endogenous Argonaute-bound RNA-CLIP (CLEAR-CLIP)	73, 2015
Quick cross-linking ligation and sequencing of hybrids (qCLASH)	74, 2018
Argonaute 2 enhanced cross-linking ligation and immunoprecipitation (AGO2-eCLIP)	75, 2023
Cross-linking and immunoprecipitation followed by intermolecular ligation of endogenous RNAs bound to Argonaute and high-throughput sequencing (CIMERA-seq)	76, 2024

^*a*^ List of published AGO-CLIP methods that include a miRNA:target RNA intermolecular ligation step followed by high-throughput sequencing.

In this minireview, we focus on the emerging evidence for miRNA sequestration by viral transcripts and for the de-repression of host target genes downstream of this miRNA regulation during viral infection, using examples identified by AGO-CLIP + ligation methods in which genome-wide miRNA: target interactions can be captured. The concept of viral transcripts functioning to sequester host miRNAs in infected cells, with potential functional impact on host transcripts bearing the same miRNA seed sequence, has yet to be extensively investigated. We posit that this miRNA regulatory mechanism could be more widely shared in viral biology and that the recent deployment of strategies allowing genome-wide profiling of ligated AGO miRNA:transcript interactions can be exploited to address this question.

## miRNA SEQUESTRATION IN HUMAN miRNA: VIRAL TRANSCRIPT INTERACTIONS

**Fig 2:**
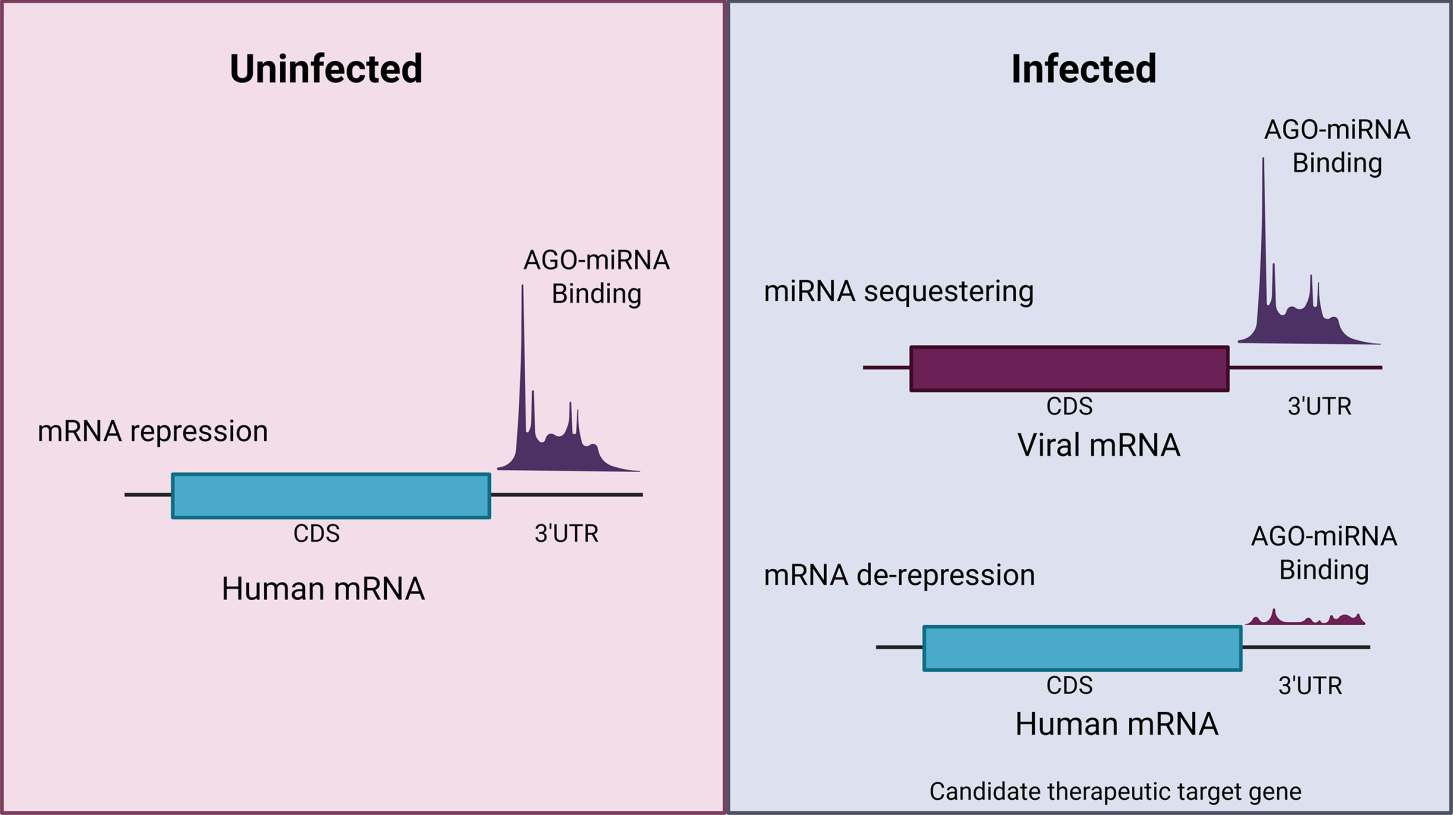
Human miRNA sequestering by viral transcripts and host mRNA target de-repression. Illustration of AGO-miRNA complexes binding to mRNA 3’UTRs in uninfected vs. infected conditions. In uninfected conditions, AGO-miRNA complex binds to cognate human 3’UTRs to repress gene expression. During infection, AGO-miRNA complexes are sequestered by complementary viral transcripts, leading to decreased binding to human mRNA 3’UTRs and de-repression of endogenous gene targets.

Human cellular miRNAs are understood to bind and post-transcriptionally regulate viral transcripts and, in many instances, to impact viral replication. Analysis of direct host miRNA: viral RNA target binding shows that human miRNA binding to viral transcripts reduces endogenous target binding, causing target de-repression, in multiple human viral infections including hepatitis C virus (HCV), hepatitis B virus (HBV), severe acute respiratory syndrome coronavirus 2 (SARS-CoV-2), and respiratory syncytial virus (RSV) (Table 1) (Fig. 2). Host miRNA sequestration by binding to viral transcripts is differentiated from viral-mediated host miRNA degradation (miRDE) by the fact that host miRNA levels are not decreased but are effectively removed from binding to host RNA targets with resulting de-repression of host transcripts.

### miR-122 and hepatitis C virus

Hepatitis C virus (HCV) is a human RNA virus that spreads through blood contact (77). HCV infection occurs in the liver, and without antiviral treatment, HCV infection persists, causing chronic liver diseases such as cirrhosis or cancer in ~70% of individuals (77–79). MiR-122 is a liver tissue-specific miRNA encoded on chromosome 18 (80) that is highly abundant in liver tissues (7). In 2005, researchers observed that certain liver and non-liver tissue-derived cell lines could propagate HCV, whereas others could not (16). Investigators tested whether miR-122 expression in the liver had a functional impact on HCV replication, using human cell lines that either expressed or did not express miR-122 (16). This work revealed that viable infection with HCV cannot occur in cells that do not express miR-122. Furthermore, two predicted binding sites for miR-122 were identified within the HCV genome, an 8-mer seed match within the 5’ non-coding region of HCV and a 7-mer within the 3’ non-coding region (16). The miR-122:HCV interaction was the first report of miRNA:viral transcript binding and functioning in a cell type-specific manner. These findings spurred research aimed at better understanding the relationship between miR-122 and HCV and more broadly to the discoveries of other host miRNA:viral target gene interactions. Cells that were transfected with 2’O methylated miR-122 inhibitors to inhibit miR-122 binding showed reduced HCV replication (16). Point mutations within the miR-122 binding sites resulted in less viral RNA and replication. Work by multiple investigators has demonstrated that miR-122 binds to the 5’ non-coding end of the HCV RNA genome (which is also a template for translation) and acts unconventionally to stabilize the RNA, enhance translation, and mediate HCV viral RNA replication (16).

miR-122 binding by HCV transcripts offers a particularly well-defined example of the biological impact of sequestering of host miRNAs by viral transcripts (56). Although miR-122 binding to HCV viral RNA has direct effects that upregulate HCV RNA abundance and translation, the global de-repression of host miR-122 targets including prolyl 4-hydroxylase alpha1 (P4HA1), pyruvate kinase m2 (PKM2), and mannan-binding lectin serine protease 1 (MASP1), by viral sequestration of miR-122 provides a cellular environment fertile for liver fibrosis and the long-term oncogenic potential of HCV (56, 81). Subsequent investigations using an AGO-CLIP approach (HITS-CLIP) both confirmed the previously identified miR-122:5’ UTR HCV RNA interaction and revealed a global view of AGO binding to both the HCV and human genomes (56). Investigators also knocked out Drosha to inhibit global miRNA processing, then expressed miR-122 mimics to demonstrate that AGO binding to HCV RNA is mediated by miR-122 using the HITS-CLIP method. Although many human transcripts in the data set included miR-122 seed binding sites, researchers reported an unanticipated decline in AGO binding to human genes with miR-122 seed binding sites in infected compared with uninfected samples (56). This finding led to the hypothesis that HCV might act as a miR-122 binding sponge and reduce AGO/miR-122 binding to cellular miR-122 mRNA targets during infection. This phenomenon was referred to as “HCV-induced miR-122 sequestration”; miR-122 binding to HCV transcripts increases during infection while host transcript binding of miR-122 decreases (56). Ultimately, the decline in AGO:miR-122 binding to human mRNAs results in miR-122 target de-repression, with an increase in the abundance and translation of host transcripts containing miR-122 seed-binding regions (56). Global miR-122 target de-repression was reported in both HCV-infected Huh-7.5 cells and human liver (56). The authors went on to show that although miR-122 binding can stabilize and promote translation of the HCV genomic RNA, this sequestration of miR-122 also de-repressed host miR-122 targets and negatively impacted the secreted virus and percent infected cells. It remains to be determined whether sequestering of host mir-122 by HCV with consequent de-repression of mir-122 targets might relate to the observed requirement for host expression of mir-122 to enable efficient HCV propagation. Additional analysis of the translation and functions of de-repressed host genes might help shed light on this relationship (56).

A miR-122 knockout mouse line was found to exhibit progressive liver inflammation and spontaneous high-grade hepatic carcinoma, indicating potential outcomes of severe global miR-122 de-repression. The capacity, specifically, for miR-122 binding by a viral transcript to mediate such consequences from the depression of host mir-122 targets was supported by a study finding that miR-122 sequestration by the integration of a portion of HBV transcript containing binding sites for miR-122 resulted in upregulated epithelial-mesenchymal transition (EMT) (82, 83), enhanced cell migration and invasion in hepatic cells, and liver damage in a mouse model. Consistently, *in vivo* treatment with miR-122 antagonist in a primate model resulted in a long-lasting suppression of HCV viremia and a long-lasting reduction of disease symptoms (81). In addition, in HCV engineered to be independent of miR-122 binding, sequestration of miR-122 could produce slight negative effects on secreted virus and the percent of infected cells. Collectively, these results suggest not only that miR-122 sequestration by HCV may confer a slightly less pro-viral cellular environment but also that this effect may be a small trade-off for the highly beneficial overall role miR-122 plays directly in the viral reproductive life cycle. Accumulated evidence for the biological importance of miR-122:HCV viral RNA interactions in the viral lifecycle and host outcomes led to the development of the first miRNA antagonist as a treatment for human viral infection. A locked nucleic acid anti-miR-122 oligonucleotide therapy, Miravirsen, exhibited dose-dependent reductions in patient HCV RNA levels (84). Although potentially a fertile ground for treatments that have been reviewed elsewhere, this therapeutic approach in the case of HCV was superseded by a developed small molecule (84).

Covalent ligation of endogenous Argonaute-bound RNAs (CLEAR)-cross-linking immunoprecipitation (CLIP) is another early AGO-CLIP + ligation method developed subsequent to CLASH (73). CLEAR-CLIP methodology is similar to CLASH but involves the IP of all four endogenous human AGO proteins using a Pan-AGO antibody instead of tagged AGO1 (73). CLEAR-CLIP was the first AGO-CLIP + ligation method used to investigate human miRNA:viral target interactions. In 2016, investigators from the previously described miR-122 HCV HITS-CLIP study 2015 (56) published a study describing host miRNA binding to viral transcripts with 15 different viruses by CLEAR-CLIP (51). Using miR-122 binding to HCV as an experimentally validated benchmark, they confirmed by CLEAR-CLIP that miR-122 directly interacts with the 5’UTR, NS5B region, and within the 3’UTR of HCV in human Huh-7.5 hepatoma cells (51). Note that a CLEAR-CLIP approach failed to detect the miR-122:HBV interaction (51). Generally, although the methods to evaluate chimeric RNA interactions continue to improve in efficiency, failure to see an interaction would not necessarily mean that the interaction does not occur. The real advantage of the chimeric approaches is not in their sensitivity *per se*, but in their genome-wide view and discovery-based detection of unsuspected interactions. Additionally, CLEAR-CLIP revealed miR-181 and miR-320 binding to HCV transcripts within the envelope region (51). This study further described another example of miR-17 sequestering by pestiviruses and functional de-repression of miR-17 host mRNA targets. Interestingly, miR-17 and let-7 miRNA binding has been shown to act similarly to enhance pestivirus RNA stability and translation (51, 85). MiR-17 and let-7 share conserved binding sites in pestiviruses including bovine viral diarrhea virus (BVDV), classical swine fever virus (CSFV), and Borna disease virus (BDV) (51, 85). However, miR-17, but not let-7, is sequestered by BVDV transcripts, which results in de-repression of host miR-17 mRNA targets (51).

### miR-15 and severe acute respiratory syndrome coronavirus 2

Severe acute respiratory syndrome coronavirus 2 (SARS-CoV-2) is a human respiratory betacoronavirus that causes coronavirus disease 2019 (COVID-19) (86, 87). SARS-CoV-2 infection caused a global pandemic in 2020 and >7 million deaths as of 2024 (88, 89). In 2023, researchers used CLEAR-CLIP to investigate miRNA:viral RNA target binding interactions in two cell lines, VeroE6 (African green-monkey kidney epithelial) and A549hACE2 (human lung epithelial), infected with the Danish ancestral isolate (DK-AHH1) strain of SARS-CoV-2 (58). Six specific AGO/miRNA binding sites were identified to be predominantly bound on SARS-CoV-2 RNA (58). The functional importance of these specific miRNA binding interactions to SARS-CoV-2 replication was tested by altering the miRNA seed binding site regions to generate either loss-of-binding or gain-of-binding viral mutants. Following mutagenesis, there was decreased or increased miRNA binding in loss-of-binding and gain-of-binding mutants, respectively. MiR-15a-5p and miR-17-5p were among the top miRNAs binding SARS-CoV-2 RNA in both VeroE6 and A549hACE2 cells. However, upon SARS-CoV-2 miRNA binding site mutagenesis and AGO2 knockdown, no major differences in SARS-CoV-2 RNA levels or viral replication were observed, although more subtle alterations in viral life cycle could not be excluded. The investigators did identify, however, that miRNA binding to host transcripts was altered by the competing miRNA binding to viral RNAs. The use of CLEAR-CLIP allowed the investigators to detect this altered target binding despite the fact that the total levels of the involved miRNAs, miR-15a-5p and miR-17-5p, were not appreciably altered by viral infection. This lack of compensatory upregulation in miR-15 expression, despite viral RNA binding in the infected host cells, impacted cellular mRNA targets of miR-15, resulting in decreased miR-15 binding to cellular RNA transcripts in infected samples relative to mock infection. Transcriptomics revealed that cellular mRNA targets for miR-15a-5p with 3’UTR miRNA binding sites were globally upregulated in SARS-CoV-2 infected samples relative to mock infection, consistent with functional target de-repression and target transcript stabilization due to sequestering of miR-15 by viral RNA and less binding to cellular mRNAs (58). Several host mRNA targets of the miR-15 family which are de-repressed have been reported to restrict SARS-CoV-2 infection and are involved in T-cell cycle progression, survival, and T-cell subset differentiation (58, 90).

### miR-26/27 and respiratory syncytial virus

Respiratory syncytial virus (RSV) is a human orthopneumovirus (91). RSV primarily infects the respiratory epithelium, causing common cold in many adults and severe lower respiratory tract illness in young children and adults aged 60 and above (92, 93). According to the CDC, there are ~60,000–160,000 annual RSV-related hospitalizations (94). Vaccines are available for adults aged 60 and above; however, there is no direct treatment for RSV infection (94). In a 2024 study, researchers applied an optimized version of the CLEAR-CLIP approach to identify miRNA: RNA target interactions during RSV infection in the human lung epithelial cell line A549 (59). Earlier work using CLEAR-CLIP to generate human-viral chimeras had revealed AGO binding to the RSV genome but had insufficient read depth to characterize the miRNA-containing chimeric RNAs (51). However, the recently optimized CLEAR-CLIP method improved read depth (59) to demonstrate through analysis of chimeric reads that human miRNAs, miR-26 and miR-27, bind to specific seed-matched sites within the RSV M transcript during infection. The RSV M codes for the viral matrix (M) protein (59). Despite binding to the M transcript, the investigators did not observe specific functional effects of miR-26 or miR-27 binding on M gene expression or a direct impact on RSV replication (59). Instead, RSV infection was observed to result in a significant decrease in miR-27 interactions with host transcripts. The authors postulated miRNA sequestering by RSV transcripts and de-repression of miR-27 and miR-26 host cell targets, as there was increased expression of some miR-27 and miR-26 human transcripts in infected samples relative to mock by RNA sequencing (59). In human cells, miR-27 is involved in metabolism and cell cycle regulation, and miR-26 is implicated in the antiviral immune response to RSV (59). De-repressed targets of miR-27 included nuclear receptor coactivator 7 (NCOA7), which is involved in metabolism, and ph domain and leucine-rich repeat protein phosphatase 2 (PHLPP2) and cyclin g1 (CCNG1), which are associated with cell cycle regulation (59); 2’−5’-oligoadenylate synthetase 2 (OAS2) and DEAD-box helicase 3 X-linked (DDX3X) were de-repressed miR-26 target transcripts (59). DDX3X has been reported as a requirement for RSV virulence (59). Human RNA target transcripts for miR-26 and miR-27 were confirmed based on seed binding sites within the 3’UTR and by RNA-sequencing analysis following treatment with miR-26 or miR-27 mimics and inhibitors (59). The study reported that RSV infection did not lead to a global de-repression of all miR-27 and miR-26 targets but was dependent on the specific interaction and expression of the targeted transcript (59). Although miR-27 interactions were specifically reduced upon infection, the opposite was observed for some miR-26 interactions. These results highlight the fact that individual host cell miRNA:target RNA interactions may be more or less susceptible to viral sequestration-based inhibition, for reasons that remain to be clarified.

## CONCLUSION

**Fig 3:**
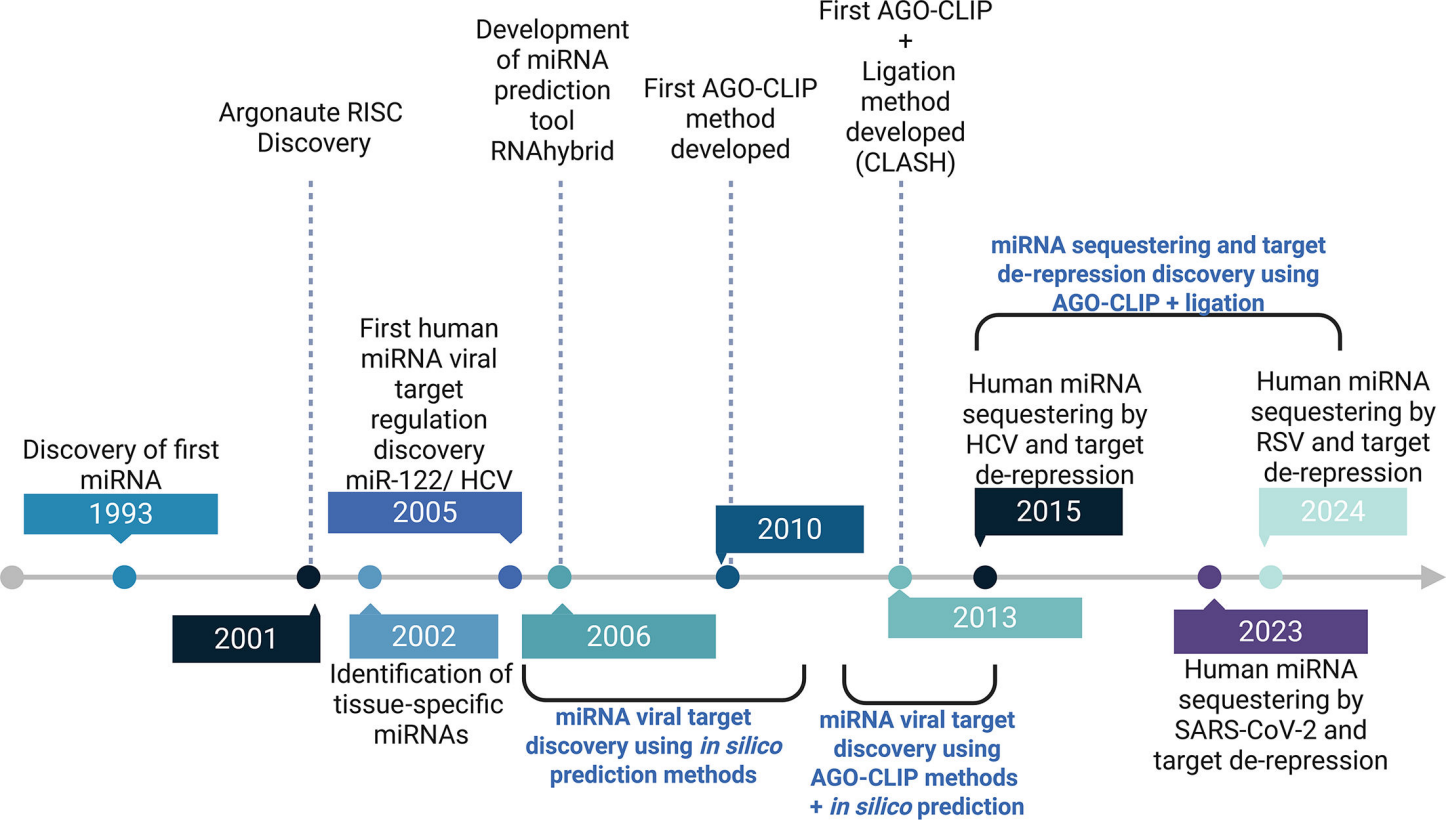
Timeline of key discoveries related to miRNA:viral target interactions. This timeline describes key advances in miRNA-target analysis tools and technology leading to the discovery of miRNA:viral target interactions and cellular miRNA sequestering by viral transcripts and target de-repression using AGO-CLIP, AGO-CLIP + ligation, and RNA-seq.

Since the foundational discovery of functional interaction between liver-specific miRNA, miR-122, and the HCV 5’UTR, many human and animal miRNA:viral RNA target interactions have been described to establish that cellular miRNAs can regulate both endogenous and viral transcripts (Fig. 3). Although miR-122 activates HCV translation and replication, other cellular miRNAs function to promote viral latency and immune system evasion by inhibiting viral replication or appear to have no direct detected impact on viral infection status. Early standard molecular approaches for validating miRNA:viral target interactions and functional importance in HCV, HIV, IAV, and KSHV included *in silico* binding predictions, seed binding site mutagenesis, translation reporter assays, and mimic or LNA inhibitor transfections. However, the utility of these methods alone has been typically limited to a select group of single miRNAs of interest, whereas hundreds of cellular miRNAs might potentially interact with viral transcripts. AGO-CLIP provided an advance in identifying global AGO binding to host and viral transcripts that was followed by predicting which miRNAs mediate specific AGO binding based on potential seed pairings. Different versions of AGO-CLIP uncovered miRNA target interactions in EBV, HSV-1, HSV-2, and HCV infection, providing insights into miRNA-mediated cellular response mechanisms and potential antiviral therapeutic targets. Additionally, when AGO-CLIP was used to study global AGO binding to HCV transcripts, decreased binding to human mRNAs in infected samples was observed, providing evidence that HCV RNA might physically sequester miR-122 from endogenous mRNA targets. Further, total RNA-sequencing revealed increased expression, or de-repression, of cellular transcripts with 3’UTR miR-122 seed binding sites, confirming the biology of miRNA sequestering and demonstrating the functional impact on cellular miRNA function by viral infection.

The functional importance of miR-122 on HCV RNA replication provided a benchmark to show that AGO-CLIP + ligation can provide a host and viral transcriptome-wide overview of direct unambiguous cellular miRNA and viral target interactions. However, as with SARS-CoV-2 and RSV, cellular miRNA binding to viral transcripts may not always serve to directly regulate viral replication. RNA sequencing in addition to AGO-CLIP or AGO-CLIP + ligation has shown that miRNA sequestering by HCV, SARS-CoV-2, and RSV transcripts can also lead to de-repression of some cellular miRNA targets, which can have significant biological effects on multiple cellular pathways in response to viral infection. Early selected and more recent global approaches allowing direct detection of genome-wide interactions clarify that miRNAs can have diverse functional roles in regulating host cellular and viral genes during infection. AGO-CLIP + ligation technologies are not limited to a select few miRNAs of interest and can instead capture the broad miRNA:target interactome. These approaches are now revealing how the cellular function of host miRNAs might be altered in response to viral infection due to sequestering by viral transcripts, which can result in the de-repression of host target genes. Improved genome-wide understanding offers an opportunity to revisit prior work and study miRNA sequestering and endogenous target depression (e.g., Table 1 interactions)(Fig. 2). This presents a new potential avenue to understand viral:host miRNA-mediated mechanisms of regulation and appreciate response pathways that can be targeted to block human viruses for prevention and treatment of chronic viral-related diseases such as cancer and neurodegeneration.
